# Group prenatal care: effectiveness and challenges to implementation

**DOI:** 10.11606/s1518-8787.2019053001303

**Published:** 2019-09-18

**Authors:** Zafiro Andrade-Romo, Ileana B. Heredia-Pi, Evelyn Fuentes-Rivera, Jacqueline Alcalde-Rabanal, Lourdes Bravo Bolaños Cacho, Laurie Jurkiewicz, Blair G. Darney

**Affiliations:** I Instituto Nacional de Salud Pública. Centro de Investigación en Sistemas de Salud. Cuernavaca, MOR, México; II University of California San Francisco. San Francisco General Hospital. Department of ObGyn & Reproductive Sciences. San Francisco, CA, USA; III Oregon Health & Science University. Department of Obstetrics & Gynecology. Portland, OR, USA

**Keywords:** Atención Prenatal, Efectividad, Modelos Organizacionales, Servicios de Salud Materno-Infantil

## Abstract

Group prenatal care is an alternative model of care during pregnancy, replacing standard individual prenatal care. The model has shown maternal benefits and has been implemented in different contexts. We conducted a narrative review of the literature in relation to its effectiveness, using databases such as PubMed, EBSCO, Science Direct, Wiley Online and Springer for the period 2002 to 2018. In addition, we discussed the challenges and solutions of its implementation based on our experience in Mexico. Group prenatal care may improve prenatal knowledge and use of family planning services in the postpartum period. The model has been implemented in more than 22 countries and there are challenges to its implementation related to both supply and demand. Supply-side challenges include staff, material resources and organizational issues; demand-side challenges include recruitment and retention of participants, adaptation of material, and perceived privacy. We highlight specific solutions that can be applied in diverse health systems.

## INTRODUCTION

Prenatal care is recognized as standard of care during pregnancy. It is usually provided by individual consultations with a trained health care provider, offering a set of cost-effective interventions that improve maternal and child outcomes and reduce complications during pregnancy, childbirth and the postpartum period^1 , 2^ .

Despite these benefits, the individual care model has been criticized in the literature for long waiting times, gaps in the continuity of care, and low user satisfaction with health care personnel^3 , 4^ .

In recent years, experiences of the implementation of group care models have been reported^5^ in high income countries such as the United States^5 , 6^ , Canada^7^ , Australia^8 , 9^ , Sweden^9^ , Netherlands^9 , 10^ , some middle and low income countries such as Bangladesh, India, Iran, Nepal, several countries in Africa, Suriname, Brazil^11^ , Haiti^12^ , and Mexico^13^ .

Group prenatal care replaces individual care. All prenatal visits are conducted with the same group of eight to 12 pregnant women, who receive a clinical evaluation in the same space by one or more health care providers, who do not vary during the follow-up of six to 10 sessions, every two to four weeks^5 , 6 , 14^ . The model has three main components: clinical assessment, education, and support. Facilitators seek to establish a less hierarchical relationship with women by involving them more actively during the session^15^ . The model has a component focused on improving perinatal and postpartum education by experience-based learning and activities that require the active participation of women. These activities are guided by a curriculum, with health topics of interest during pregnancy, childbirth and postpartum. There is a key element of socialization and an element of self-evaluation of some health parameters^5^ .

The CenteringPregnancy model has been the most studied and internationally used group prenatal care model^5 , 6 , 11 , 16 , 17^ . A midwife created it in the United States in the early 1990s^15^ . It is a flexible model, but with essential elements that must be met during all sessions ( Table 1 )^18^ . Other group care models have been described^5 , 8^ ; however, most of the literature focuses on the adaptation, implementation, and evaluation of the CenteringPregnancy model.

**Table 1:** Essential elements of the CenteringPregnancy model and key points for its implementation.

Essential element	Key points
1. Health assessment occurs within the group space	The assessment area is set up to ensure privacy: position and level of the exam area, music, plants, or some kind of simple division
2. Women are involved in self-care activities	Women collect and record some health parameters such as their blood pressure or weight
3. A facilitative leadership style is used	Facilitators guide women but do not control the discussion and refer questions to the group Women share their experiences, feelings, ideas and information voluntarily Rules are established for the group Facilitators dress casually Participants sign a confidentiality agreement
4. Each session has an overall plan although the emphasis may change	Materials are used to guide and evaluate the session
5. The group is conducted in a circle	There are no observers outside the circle The space where the session takes place is private and leads to share experiences People sit in a circle in an open space The circle activities do not start until all facilitators and women are in the circle
6. Group members composition is stable, including facilitators	New women can join the group as long as there is an agreement between the members of the group Facilitators are present during the entire session There’s a plan in case a facilitator is missing If there are student supporters in the group, they are supervised and consistent throughout the sessions Women’s children are not present during circle activities
7. Group size is optimal to promote the process	The groups are between 8-12 women There is an appropriate balance between women and facilitators
8. Opportunity for socializing within the group is provided	There is a free time in which women can socialize with each other
9. There is on-going evaluation of outcomes	Outcomes, model fidelity and sustainability are reported Facilitators are constantly asking women how they feel about group prenatal care

Adapted from Rising et al.^18^

Currently, there is a worldwide interest in the model. The American College of Obstetricians and Gynecologists (ACOG) and the World Health Organization (WHO) have highlighted its potential benefits, leading to improvements in quality of care and outcomes in maternal and perinatal health in diverse populations. Both institutions have recommended the implementation of the model, encouraging research to generate reliable evidence about its effects in the short, medium, and long term^2 , 5^ .

There is scarce scientific literature in Spanish that documents the benefits of this new model and the challenges that arise during its implementation. This limitation represents a barrier to Spanish-speaking countries to access information and make decisions regarding its implementation. The objectives of this article are: 1) present a narrative synthesis in Spanish of the most recent and relevant evidence on the maternal and perinatal health outcomes of the group prenatal care model, 2) identify and summarize the challenges of implementing the model in different contexts, as well as the solutions recommended in the literature, and 3) share our experience of implementing and adapting the model to the Mexican context.

## NARRATIVE REVIEW OF THE LITERATURE ABOUT THE EFFECTIVENESS OF GROUP PRENATAL CARE

From January 23, 2018, to July 27, 2018, we conducted a narrative or classical review^20^ of the most recent literature. The search period included 2002 to 2018. We used the following search terms in Spanish: atención prenatal en grupo, control prenatal en grupo, embarazo and atención en grupo. And in English: group antenatal care, group prenatal care, centering pregnancy, pregnancy, pregnant women, prenatal education, prenatal care, antenatal care, antenatal control, prenatal control, antenatal visit, pregnancy care and group visit or group care. We used databases (PubMed, EBSCO, Science Direct, Wiley Online, the publisher Springer) and registry pages of systematic reviews such as The Cochrane Library and Prospero. Because of our first objective, we gave priority to systematic reviews, meta-analyses and randomized clinical trials that have comparatively evaluated individual prenatal care and group models in different maternal and perinatal health outcomes^6 , 16 , 17 , 21^ ( Table 2 ).

**Table 2:** Neonatal and maternal outcomes of group prenatal care compared to individual prenatal care.

Results	Better results with group prenatal care	Inconclusive results
Neonatal		

Preterm Birth		Mazzoni, 2017; Carter, 2016; Catling, 2015; Tilden, 2014
Low birth weight	Ford, 2002^a,b^	Mazzoni, 2017; Carter, 2016; Catling, 2015; Tilden, 2014
Size for gestational age	Ickovics, 2016^a^	Catling, 2015
Intensive Care Units admission rate		Mazzoni, 2017; Carter, 2016; Catling, 2015
Neonatal mortality		Catling, 2015

Maternal		

Weight gain during pregnancy		Mazzoni, 2017
Prenatal knowledge	Mazzoni, 2017; Tilden, 2014	
Satisfaction	Kennedy, 2011^a^	Mazzoni, 2017; Tilden, 2014
Increased social support		Kennedy, 2011^a^; Ickovicks, 2011^a^
Stress		Mazzoni, 2017; Catling, 2015
Depression		Mazzoni, 2017; Catling, 2015
Prenatal visits attendance	Kennedy, 2011^a^	Ickovics, 2016^a^
Cesarean section rate		Tilden, 2014
Initiation of Breastfeeding	Tilden, 2014	Mazzoni, 2017; Carter, 2016; Catling, 2015
Breastfeeding continuation	Tilden, 2014	Mazzoni, 2017
Family planning during the postpartum period	Mazzoni, 2017	
Planned and unplanned pregnancy in less than 1 year		Ford, 2002^a,b^
Need for medication for gestational diabetes	Byerley, 2017^a^	
Insulin requirement	Byerley, 2017^a^	

NOTE: The table focuses mainly on the results of systematic reviews, most recent meta-analyses and clinical trials. The inconclusive results are because some studies report better outcomes in group prenatal care and others report no difference.

^a^ Clinical Trials

^b^ Group prenatal care model different from CenteringPregnancy

Evidence showed better maternal outcomes in terms of the knowledge that pregnant women acquire during group prenatal care, nutrition, breastfeeding, changes during pregnancy, family planning and substance abuse^6 , 17^ ; as well as increased use of family planning services in the postpartum period^6^ and reduced use of medication in women with gestational diabetes^26^ , compared to pregnant women in individual care ( Tables 2 and 3 ).

**Table 3:** Reviews and clinical trials: outcomes of interest of women in group prenatal care.

Author and year of the study	Type of study	Sample size (n of sample in group prenatal care)	Population	Outcomes of interest of women in group prenatal care
Mazzoni, 2017	Literature Review	X	X	Greater knowledge and better postpartum family planning
Byerley, 2017	Systematic literature review	X	X	Lower medication requirement in women with gestational diabetes (30.2 vs. 42.1%; p = 0.009). Women with gestational diabetes who required insulin needed less than half of the dose (26% versus 63%, p < 0.001)
Tilden, 2014	Literature Review	5,650 (2,080) of the 9 final studies analysed	X	More appropiate initiation of breastfeeding (66.5% vs. 54.6%) (p = 0.001) and longer duration of breastfeeding (94.3% vs. 86.7%) (p = 0.001)
Ickovics, 2016	Randomized group-controlled clinical trial	1,148 (573)	Adolescents aged 14-21, < 24 weeks of gestational age	Less possibilities of having children with a small size for gestational age (<10th percentile) (11.0% vs 15.8%; OR 0.66; 95% IC 0.44-0.99)
Kennedy, 2011	Randomized clinical trial	322 (162)	Military personnel over 18 years old, < 16 weeks of gestational age	Six times more likely to receive better prenatal care. Greater satisfaction with their health care (p < 0.001). Higher average number of prenatal visits (10.31 visits vs. 8.56) and, of those in group prenatal care, only 12.9% had fewer than 9 visits vs. 46.7% of those in individual care p < 0.005, OR:5.92 IC 3.21-10.91)
Ford, 2002	Randomized clinical trial	282 (165)	Adolescents aged 13-21	Women in group prenatal care had lower rates of low birth weight (6.6% vs. 12.5%, p 0.08)

For other maternal outcomes, such as the initiation of breastfeeding^6 , 16 , 17 , 21^ , the rate of cesarean section^17^ or attendance to prenatal visits^23 , 25^ (among others), the available evidence was not always conclusive ( Table 2 ). The results varied, depending on the characteristics of the population^5 , 6 , 26^ ( Table 3 ).

The most studied perinatal results are preterm childbirth^6 , 16 , 17 , 21^ , low birth weight^6 , 16 , 17 , 21 , 22^ , Intensive Care Unit admission^6 , 16 , 21^ , size for gestational age^21 , 23^ and neonatal mortality^21^ . Results were inconclusive ( Table 2 ), but no studies have reported that the model has harmful outcomes either for the mother or the newborn^16^ .

The group model achieved greater effectiveness for particular outcomes (knowledge, family planning, and use of services). Because the model is women-focused, these are perhaps the outcomes most likely to be achieved. Others require more structural changes in the health system and social determinants, beyond just a change in the model of care. Future evaluations should strengthen evidence about the effects on satisfaction, self-efficacy, empowerment, and improvements in the experience during pregnancy. According to the principles of client-centered care models, these are more immediate results to be achieved^27^ .

## EVIDENCE ABOUT IMPLEMENTATION OF GROUP PRENATAL CARE MODELS IN DIFFERENT CONTEXTS: CHALLENGES AND POTENTIAL SOLUTIONS

Implementation of the group prenatal care model involves challenges that can be analyzed from the user or the provider’s perspective.

From the provider’s perspective, challenges are those related to the personnel who will implement the model^6^ , material resources and physical inputs^5 , 6 , 17^ and organizational challenges, specific to national institutions or regulations for the provision of health services^13^ ( Table 4 ). These challenges have a potential impact on acceptability of the model by health care providers^6^ . From the provider’s perspective, the greatest challenges involve infrastructural issues of the health care services. Although physical spaces requirements are crucial for the implementation of the model, feasible alternatives are available^12^ .

**Table 4:** Challenges, recommendations and potential solutions for implementing the group prenatal care model in different contexts.

Challenges			Recommendations and potential solutions and experience in Mexico	Essential element involved (Table 1)
“Providers”	Health care personnel to implement the model	Profile	Multidisciplinary team: may include professional midwives, medical staff, nursing staff, health educator. It is important that they feel comfortable with a change of mentality and have a positive attitude to implement a new model.	No explicitly identified
			Medical, nursing and health promotion staff.	
		Number	One, two or three facilitators per group.	7
			Two to three facilitators per group.	
		Continuity of care	Same team of facilitators in each session.	6
			Train two teams of facilitators in the same health center who have the capacity to follow-up with women if necessary.	
		Training (cost and logistics)	Funding and support from the people in charge of the health care services, for local training.	No explicitly identified
			Schedule trainings with multiple health centers. Build and train of local “trainers” who can replicate the workshops in the country.	
		To achieve a facilitating style and a more horizontal relationship between health care personnel and the user	Reinforce during the training that information should be facilitated among the entire group rather than solely provided by health care personnel. Facilitators guide but do not control the discussion. Facilitators should avoid clothing that denotes hierarchy (e.g., lab coats). Women are involved in measuring some health parameters.	2, 3, 5, 8, 9
			Ensure during all the sessions, at least in the first group, a person who is helping to reinforce the new dynamic and at the end of the session share with the facilitators what happened during the session and what can be improved.	
	Material resources and physical inputs	Physical space	Look for a semi-private space in the clinic that allows at the same time the presence of 10-12 people in a circle and that in the same space but behind the circle a space can be installed to carry out physical examinations.	1, 5, 8
			Use waiting rooms or similar rooms during not busy times, an open space under the trees, parking lot, the house of one person of the group, a tent and preferably early in the morning or in the afternoon to avoid high temperatures. Search for community places such as churches or other spaces.	
			Find a place close to the clinic (government space, church, public center) that is semi-private, free, that allows a short transfer of the health care personnel and the material to be used, and that is accessible to women.	
		Adaptation of the material and recurrent costs	Adapt the material considering aspects such as socioeconomic status, race, religion, language and culture.	4
			Use local literature, experts, and locals to adapt the material.	
			Work on the adaptation of the material with experts and a multidisciplinary team that knows the population and think about activities that do not require regular purchases. Identify recurring material (e.g., sheets of paper, batteries for sphygmomanometers) and make a plan to apply for support at state or federal level.	
	Organizational	Management and coordination within the units / clinics	Involve clinic managers directly in the project emphasizing the importance of support and knowledge of all clinical staff to solve unexpected situations and flexibility during the days needed to conduct group prenatal care.	No explicitly identified
		National regulations	Adapt materials following national regulations, designing a curriculum taking into consideration the topics referred in the Mexican Official Norms that pregnant women should see during their prenatal consultations; but being flexible enough to include emerging topics of interest to women.	4
Users	Users of the model	Recruitment	Recruit participants with similar weeks of gestation.	7
			It is important that all clinical staff is aware of the benefits of the model so that they can invite women to participate and that people who have the initial contact with pregnant women are trained into how to recruit women. The person recruiting women should be a health worker who women trust. Print flyers or materials to help explain the model during the recruitment process. If possible, include the partner during the recruitment process.	
			Identify places with enough volume of pregnant women.	
		Retention	Retention of participants with similar weeks of gestation.	6
			Electronic reminders of the next session.	
		Language and educational level of users	Adapt materials to the educational level and language of the key population.	4
			Have trained facilitators who speak the language of the population. Carry out activities that do not require women to read but only talk, also activities that include songs from the community. Conduct the self-evaluation always with the support of the facilitators.	
			Identify simple activities and materials and adapt the materials with the help of experts and a multidisciplinary team that knows the population, adapt popular games of the place.	
		Privacy	Use music and have a space for clinical exams behind the circle a little far from it.	1
			Use a screen or curtain to separate the clinical assessment space.	
			Use a screen to separate the clinical review space.	
		For women to adhere to the rules established in the group (schedule, bring or not companions)	Make group rules clear in the first session and allow women to change rules that do not suit their needs.	6,7
			In some cases allow companions during all sessions, including children.	
			Flexibility to start the session 15-20 minutes later, or to allow some women to come into the session and incorporate them into the activities. Allow them to bring their companions even if it is not consistent, especially their partners, since they may have trouble being granted permission at work to attend all sessions.	

NOTE: In the column of recommendations, potential solutions and experience in Mexico, we describe in white the recommendation to implement the model, in gray the solution described in the literature and in blue our experience in Mexico.

Group prenatal care has been implemented in diverse populations in the United States: African-American women^5 , 6 , 17 , 22^ , military^5 , 6 , 16 , 17^ , and some populations that may be relevant to countries in Latin America such as: adolescents^5 , 6 , 26^ , Latina or Hispanic^5 , 6 , 16^ , women with diabetes^5 , 6 , 26^ , and low-income women^5 , 6 , 11 , 16 , 26^ . From the users’ perspective, challenges to implementation are less common, but include: women’s willingness to participate in a new model of care, attendance at medical consultations^6^ , patients’ language and education level, and patients’ adjusting their schedules in order to participate in the group (considering that the group sessions last longer than an individual consultation)^12^ . Other reported challenges include women’s privacy concerns due to the group nature of care^5^ . The user-centered approach of the group care model may explain why so few challenges have been identified from a users’ perspective. This type of health care has also been linked to a positive perception of rights by the users, influencing the acceptability of the model^27^ .

Some of the challenges noted in the literature may depend on the context or culture, for which very specific solutions have been proposed ( Table 4 ). However, discussion and dissemination of challenges and solutions among stakeholders, both clinical and academic, and with decision-makers, may be useful for future efforts to implement the model in Latin America.

## THE MEXICAN EXPERIENCE DURING THE ADAPTATION AND IMPLEMENTATION OF A GROUP PRENATAL CARE MODEL

During 2016-2018, researchers at the *Instituto Nacional de Salud Pública de México* (INSP - National Institute of Public Health of Mexico), adapted and implemented the CenteringPregnancy model in the public sector of the Mexican health system. As in many countries, this sector faces particular challenges, especially in infrastructure, financing, and organization.

We summarize our experience of adapting and implementing the model in Table 5 . A more detailed description of the adaptation process can be found in a previous article^13^ . Two challenges and their respective solutions or recommendations are highlighted ( Tables 4 and 5 )^28^ .

**Table 5:** Experience of adaptation and implementation of a Group Prenatal Care model in Mexico, 2016-2018.

Adaptation and implementation of the model in Mexico	Description		
Phases of the feasibility study	Consisted of six phases carried out from June 2016 to August 2018. 1. **Involvement of the Department of Health and training of the Mexican team** . We negotiated the implementation of the model with decision makers from two Mexican states (Morelos and Hidalgo). Simultaneously, two members of the research team were trained as facilitators in an intensive CenteringPregnancy workshop in the United States. During this and subsequent phases, we had the support of an international expert in the model. 2. **Adaptation of the Centering Pregnancy model to the Mexican context.** We created a curriculum based on the guidelines of prenatal care by the Department of Health, the perception of pregnant women and experts in the subject of maternal health. We also adapted material resources and physical inputs. 3. **Selection of participating places.** We did a pre-feasibility study in several first-level care centers in the states of Morelos and Hidalgo, identifying structural elements (number of clinics, health staff, available spaces, etc.) as well as the volume women in prenatal care (number of prenatal visits in the last year). Considering this information and support from the international expert, we selected the health centers where the model was implemented. 4. **Training of health staff at health centers.** We trained health providers (nurses, medical and social workers) to take on the role of facilitators of the model. This phase also helped to consolidate two team members as model trainers. 5. **Pilot Study.** In this phase we began the implementation of the model in two sites, one in each state. We provided the necessary material for implementation and gave advice for the initial recruitment of women. The research team provided direct support in solving problems. We adapted the processes, the instruments, the topics, the methodology and the logistical aspects together with the international expert. 6. **Implementation.** Considering all the adaptations of the previous phases, we extended the implementation of the model to all the selected sites.		
States where we implemented the model	The selected states were: Morelos and Hidalgo. The Sanitary Jurisdictions*: Morelos- Jurisdiction 1. Hidalgo- Jurisdiction 3.* The implementation places belong to the Department of Health (Public Health Services) that provides services to the population that does not have any kind of social security, the most vulnerable population of the country that does not have a job in the formal sector of the economy.		
Number and places where we implemented the model	Implementation places in Morelos (3): Centros de Salud Acatlipa, Temixco and Emiliano Zapata Implementation places in Hidalgo (1): Centro de Salud de Tula		
Number and profile of trained facilitators per location	A total of 23 health care providers were trained during the study (3 trainings of 2 days each), of which 15 participated as group facilitators in different centers:		
	Location	Total of facilitators	Facilitators’ profile
	Acatlipa, Morelos	3	2 medical doctors and 1 nurse
	Temixco, Morelos	2	1 medical doctor and 1 nurse
	Emiliano Zapata, Morelos	5	2 medical doctors, 2 nurses and 1 health promoter (Degree in Psychology)
	Tula, Hidalgo	5	2 medical doctors, 2 nurses and 1 social worker
Number of groups implemented	A total of 11 groups were conducted in both states (seven in Morelos and four in Hidalgo): • Morelos: Acatlipa (3 groups), Temixco (2 groups), Emiliano Zapata (2 groups) • Hidalgo: Tula (4 groups)		
Number of pregnant women recruited	The total number of pregnant women recruited for the 11 groups was 142.		
Number of pregnant women who completed the model	76.0% (108) completed group prenatal care.		
Gestational age at the beginning of the model	Average gestational age at the beginning of prenatal group care: 23.0 weeks gestation. Range: 21.9 - 24.1.		
Average number of prenatal visits	5.5 visits per woman		
Percentage of women with at least 5 prenatal visits	The total number of women who complied the provisions of the Official Mexican Norm on prenatal care was 115 (81.0%).		

* Refers to a technical-administrative unit decentralized from the Public Health Services, with capacity for planning, administration, direction, operation and evaluation of resources to “provide medical care to the uninsured population, with the purpose of adequately conducting the actions of the sector in its area of influence”^28^.

The first challenge was selecting health facilities (clinics) to participate in implementing the model, which requires specific criteria. These criteria include: 1) having an adequate physical space for group care, allowing a meeting of approximately 15 people; 2) having enough health care personnel at each location so there is more than one team of facilitators trained, both for group follow-up and for recruiting participants; and 3) having clinic management and administrative support, which is key due to the time that the health care personnel has to allocate to logistics and organization activities before, during and after the sessions.

The second main challenge we want to highlight is the change of mentality about how to deliver care during pregnancy, due to the prevailing hierarchical medical culture. The presence of a research team member during the sessions enabled reinforcement of this new facilitating style and immediate feedback once the session was over. We had a research team member present as part of our feasibility study; however, monitoring implementation may not always be possible. Strategies such as intermediate reinforcement trainings during the time between the “adaptation” and “adoption” stages could be established. The first stage allows adjusting the model to the local context; the second (more difficult) refers to health care personnel and users adopting it as a learned and internalized practice.

Preliminary evidence showed implementation feasibility in Mexico, as long as a process of staggered steps is followed which can promote the buy-in of the relevant health sector authorities, training, and on-going support to participating health care personnel.

## FINAL CONSIDERATIONS

The quality and continuity of prenatal care remains a challenge in Spanish-speaking and Latin American countries^29^ , especially in vulnerable populations^30^ . Group prenatal care represents an opportunity to improve the quality of care and health outcomes for these populations: it is a user-centered model, it improves maternal health outcomes, achieves greater adherence to prenatal care, increases knowledge among pregnant women, and increases the use of postpartum family planning services.

The main approach of the model is that care is provided in a group setting with the women at the center, which motivates participation and interest of the patient. In addition, it favors a positive experience during pregnancy, with respectful care, based on a vision of rights, high level of participation and involvement of the pregnant woman during her health care encounter. Group prenatal care is an innovative and valuable approach as an alternative to the existing models.

Finally, it is necessary to develop studies with rigorous designs, and on a larger scale, both in the national context and in countries similar to ours. These studies can contribute to generate evidence to support recommendations on the implementation of this type of models and eventually evaluate not only feasibility but sustainability.
