# Supplementary material for: Group prenatal care: effectiveness and challenges to implementation
Source: Rev Saude Publica. 2019 Sep 18;53:85. doi: 10.11606/s1518-8787.2019053001303 (PMC6763281; doi:10.11606/s1518-8787.2019053001303)
Supplement: Translation

# Translation: Atención prenatal en grupo: efectividad y retos de su implementación

**Authors:** Zafiro Andrade-Romo, Ileana B. Heredia-Pi, Evelyn Fuentes-Rivera, Jacqueline Alcalde-Rabanal, Lourdes Bravo Bolaños Cacho, Laurie Jurkiewicz, Blair G. Darney

**Affiliations:** I Instituto Nacional de Salud Pública. Centro de Investigación en Sistemas de Salud. Cuernavaca, MOR, México; II University of California San Francisco. San Francisco General Hospital. Department of ObGyn & Reproductive Sciences. San Francisco, CA, USA; III Oregon Health & Science University. Department of Obstetrics & Gynecology. Portland, OR, USA

**Keywords:** Atención Prenatal, Efectividad, Modelos Organizacionales, Servicios de Salud Materno-Infantil

## Abstract

La atención prenatal en grupo es un modelo alternativo de atención durante el embarazo, que sustituye la atención prenatal individual estándar. El modelo ha mostrado beneficios maternos y se ha implementado en diferentes contextos. Llevamos a cabo una revisión narrativa de la literatura en relación a su efectividad, utilizando bases de datos como PubMed, EBSCO, Science Direct, Wiley Online y la editorial Springer, para el periodo 2002 a 2018. Adicionalmente, discutimos los retos y soluciones de su implementación desde nuestra experiencia en México. La atención prenatal en grupo puede mejorar el conocimiento prenatal y el uso de servicios de planificación familiar en el postparto. El modelo se ha implementado en más de 22 países y existen retos de su implementación desde la oferta y la demanda. Los retos desde la oferta incluyen al personal, recursos materiales y cuestiones organizacionales; desde la demanda, el reclutamiento y retención de participantes, adaptación del material y privacidad percibida. Resaltamos soluciones concretas que pueden aplicar a diversos sistemas de salud.

## INTRODUCCIÓN

La atención prenatal es reconocida como un estándar de atención durante el embarazo. Generalmente, se brinda a través de consultas individuales, con un proveedor capacitado, ofertándose un conjunto de intervenciones costo-efectivas que mejoran los resultados materno-infantiles y reducen complicaciones durante el embarazo, parto y puerperio^1 , 2^ .

A pesar de estos beneficios, el modelo de atención individual ha sido criticado en la literatura por los largos tiempos de espera, las brechas en la continuidad de la atención y la baja satisfacción de las usuarias con el trato del personal de salud^3 , 4^ .

Durante los últimos años, se han reportado experiencias sobre la implementación de modelos de atención grupal^5^ en países de ingresos altos como Estados Unidos (EEUU)^5 , 6^ , Canadá^7^ , Australia^8 , 9^ , Suecia^9^ , Países Bajos^9 , 10^ , algunos países de ingresos medios y bajos como Bangladesh, India, Irán, Nepal, varios países en África, Surinam, Brasil^11^ , Haití^12^ y México^13^ .

La atención prenatal en grupo reemplaza la atención individual. Todas las visitas prenatales se llevan a cabo con un mismo grupo de ocho a 12 gestantes, que reciben una evaluación clínica en un mismo espacio, por uno o más proveedores de salud, que no varían durante el seguimiento de seis a 10 sesiones, cada dos a cuatro semanas^5 , 6 , 14^ . El modelo tiene tres componentes principales: evaluación clínica, educación y apoyo. Los facilitadores buscan establecer una relación menos jerárquica con las mujeres, al involucrarlas más activamente durante la sesión^15^ . El modelo posee un componente centrado en mejorar la educación perinatal y del puerperio, por medio de un aprendizaje basado en experiencias y actividades que requieren una participación activa de las mujeres. Estas actividades son guiadas por un currículo, con temas de salud de interés durante el embarazo, parto y puerperio. Existe un elemento clave de socialización y un elemento de auto-evaluación de algunos parámetros de salud^5^ .

El modelo *CenteringPregnancy* ha sido el modelo de atención prenatal grupal más estudiado y utilizado internacionalmente^5 , 6 , 11 , 16 , 17^ . Fue creado por una partera en EEUU a inicios de los años 90^15^ . Es un modelo flexible, pero con elementos esenciales que deben cumplirse durante todas las sesiones ( Tabla 1 )^18^ . Se han descrito otros modelos de atención grupal^5 , 8^ ; sin embargo, la mayor parte de la literatura se enfoca en la adaptación, implementación y evaluación del modelo *CenteringPregnancy* .

**Tabla 1:** Elementos esenciales del modelo *CenteringPregnancy* y puntos clave para su implementación.

Elemento esencial	Puntos-clave
1. El examen médico se produce dentro del espacio grupal	Organizar algunos elementos de tal manera que el área destinada para el examen médico asegure la privacidad: lugar y nivel del área, música, plantas o algún tipo de división sencilla
2. Las mujeres están involucradas en actividades de auto-cuidado	Las mujeres se toman y registran algunos parámetros de salud como su presión arterial o su peso
3. Se utiliza un estilo de liderazgo facilitador	Los facilitadores guían pero no controlan la discusión y refieren las preguntas al grupo Las mujeres comparten sus experiencias, sentimientos, ideas e información de manera voluntaria Se establecen reglas para el grupo Los facilitadores se visten de manera casual Las participantes firman un acuerdo de confidencialidad
4.Cada sesión tiene un plan general aunque el énfasis puede variar	Se utilizan los materiales para guiar y evaluar la sesión
5. El grupo se lleva a cabo en un círculo	No hay observadores fuera del círculo El espacio donde se lleva a cabo la sesión es privado y lleva a compartir experiencias Las personas se sientan en círculo en un espacio abierto El inicio de las actividades del círculo no se lleva a cabo hasta que todos los facilitadores y las mujeres se encuentren en el círculo
6. Hay estabilidad en los miembros del grupo incluyendo los facilitadores	Se pueden unir nuevas mujeres al grupo siempre y cuando sea un acuerdo entre los miembros del grupo Los facilitadores están presentes durante toda la sesión Hay un plan por si algún facilitador no puede acudir Si existen estudiantes apoyando en el grupo, estos son supervisados y son consistentes a lo largo de todas las sesiones Los niños de las mujeres no están presentes durante las actividades del círculo
7. El tamaño del grupo es óptimo para promover el proceso	Los grupos tienen entre 8-12 mujeres Hay un balance apropiado entre mujeres y facilitadores
8. Se proporciona la oportunidad para la socialización con el grupo	Existe un tiempo libre en el cual las mujeres pueden socializar entre ellas
9. Hay una evaluación continua de los resultados	Hay un reporte de fidelidad, resultados en salud y sostenibilidad del grupo Los facilitadores le preguntan constantemente a las mujeres cómo se sienten con la atención prenatal en grupo

Adaptada de Rising et al.^18^

Hay un interés mundial en este modelo de atención. El Colegio Americano de Obstetras y la Organización Mundial de la Salud han resaltado sus potenciales beneficios, propiciando mejoras en la calidad de la atención y en los resultados en salud materna y perinatal en diversas poblaciones. Ambas instituciones han recomendado la implementación del modelo, incentivando el desarrollo de investigaciones que generen evidencia robusta sobre sus efectos a corto, mediano y largo plazo^2 , 5^ .

Existe poca literatura científica en español que documente los beneficios de este nuevo modelo y los retos que se originan durante su implementación^19^ . Esto representa una limitante para que países hispanohablantes puedan acceder a información y tomar decisiones en cuanto a su implementación. Los objetivos de este artículo fueron: 1) presentar una síntesis narrativa, en español, de la evidencia más reciente y relevante sobre los resultados en salud materna y perinatal del modelo de atención prenatal en grupo, 2) identificar y resumir los retos de la implementación del modelo en distintos contextos, así como las soluciones recomendadas en la literatura, y 3) compartir nuestra experiencia durante la implementación y adaptación del modelo al contexto mexicano.

## REVISIÓN NARRATIVA DE LA LITERATURA SOBRE LA EFECTIVIDAD DE LA ATENCIÓN PRENATAL EN GRUPO

Revisión narrativa o clásica^20^ de la literatura más reciente del 23 de Enero de 2018 al 27 de Julio de 2018. El periodo de búsqueda abarcó del año 2002 al año 2018. Utilizamos los términos de búsqueda: atención prenatal en grupo, control prenatal en grupo, embarazo y atención en grupo, “ *group antenatal care* ”, “ *group prenatal care* ”, “ *centering pregnancy* ”, “ *pregnancy, pregnant women, prenatal education, prenatal care, antenatal care, antenatal control, prenatal control, antenatal visit, pregnancy care and group visit or group care* ”. Se utilizaron buscadores y bases de datos como PubMed, EBSCO, Science Direct, Wiley Online, la editorial Springer y páginas de registro de revisiones sistemáticas como The Cochrane Library y Prospero. Debido a nuestro primer objetivo, dimos prioridad a revisiones sistemáticas, meta-análisis y ensayos clínicos aleatorizados que han evaluado de forma comparativa la atención prenatal individual y los modelos grupales en varios resultados de salud, tanto maternos como perinatales^6 , 16 , 17 , 21^ ( Tabla 2 ).

**Tabla 2:** Resultados neonatales y maternos de la atención prenatal en grupo comparada con la atención prenatal individual, 2002–2018.

Resultados	Mejores resultados con atención prenatal en grupo	Resultados no concluyentes
Neonatales		

Parto pretérmino		Mazzoni, 2017; Carter, 2016; Catling, 2015; Tilden, 2014
Bajo peso al nacer	Ford, 2002^a,b^	Mazzoni, 2017; Carter, 2016; Catling, 2015; Tilden, 2014
Talla para edad gestacional	Ickovics, 2016^a^	Catling, 2015
Tasa de admisión a UCI (Unidades de Cuidados Intensivos)		Mazzoni, 2017; Carter, 2016; Catling, 2015
Mortalidad neonatal		Catling, 2015

Maternos		

Aumento de peso durante el embarazo		Mazzoni, 2017
Conocimiento prenatal	Mazzoni, 2017; Tilden, 2014	
Satisfacción	Kennedy, 2011^a^	Mazzoni, 2017; Tilden, 2014
Aumento de apoyo social		Kennedy, 2011^a^; Ickovicks, 2011^a^
Estrés		Mazzoni, 2017; Catling, 2015
Depresión		Mazzoni, 2017; Catling, 2015
Asistencia a visitas prenatales	Kennedy, 2011^a^	Ickovics, 2016^a^
Tasa de cesárea		Tilden, 2014
Inicio de lactancia	Tilden, 2014	Mazzoni, 2017; Carter, 2016; Catling, 2015
Continuación de lactancia	Tilden, 2014	Mazzoni, 2017
Planificación familiar en el postparto	Mazzoni, 2017	
Embarazo planeado y no planeado en menos de 1 año		Ford, 2002^a,b^
Necesidad de medicamentos para diabetes gestacional	Byerley, 2017^a^	
Requerimiento de insulina	Byerley, 2017^a^	

NOTA: La tabla se enfoca principalmente en los resultados de las revisiones sistemáticas, meta-análisis más recientes y ensayos clínicos. Los resultados no concluyentes se deben a que en algunos estudios se reportan mejores resultados en atención prenatal en grupo y en otros no hubo diferencia.

^a^Ensayos clínicos

^b^Otro modelo de atención prenatal en grupo diferente a *CenteringPregnancy*

La evidencia disponible refirió mejores resultados maternos relacionados al conocimiento que las gestantes adquieren durante la atención prenatal grupal en temas como nutrición, lactancia, cambios durante el embarazo, planificación familiar y abuso de sustancias^6 , 17^ ; así como un mayor uso de servicios de planificación familiar en el postparto^6^ y un menor uso de medicamentos en mujeres con diabetes gestacional^26^ , comparadas con gestantes en atención individual (Tablas 2 y 3).

A pesar de que se han estudiado otros resultados maternos como inicio de lactancia^6 , 16 , 17 , 21^ , tasa de cesárea^17^ o asistencia a visitas prenatales^23 , 25^ (entre otras), la evidencia disponible no siempre fue concluyente ( Tabla 2 ). Los resultados variaron dependiendo de las características de la población^5 , 6 , 26^ ( Tabla 3 ).

**Tabla 3:** Revisiones y ensayos clínicos: resultados de interés en mujeres en atención prenatal en grupo, 2002–2018.

Autor y año del estudio	Tipo de estudio	Tamaño de la muestra (n de la muestra en atención prenatal en grupo)	Población	Resultados de interés de mujeres en atención prenatal en grupo
Mazzoni, 2017	Revisión de la literatura	X	X	Mayor conocimiento y mejor planificación familiar postparto
Byerley, 2017	Revisión sistemática de la literatura	X	X	Menor requerimiento de medicamentos en mujeres con diabetes gestacional (30.2 vs. 42.1%; p = 0.009). Aquellas mujeres con diabetes gestacional que requerían insulina necesitaron menos de la mitad de la dosis (26% versus 63%, p < 0.001).
Tilden, 2014	Revisión de la literatura	5650 (2080) de los 9 estudios finales analizados	X	Inicio de lactancia más oportuna (66.5% vs. 54.6% ) (p = 0.001) y mayor duración de lactancia (94.3% vs. 86.7%) (p = 0.001)
Ickovics, 2016	Ensayo clínico aleatorizado controlado por grupos	1148 (573)	Adolescentes de 14-21 años, < 24 semanas de gestación	Menos posibilidades de tener recién nacidos con una talla pequeña para edad gestacional (<10mo percentil) (11.0% vs 15.8%; OR: 0.66; 95% IC: 0.44-0.99)
Kennedy, 2011	Ensayo clínico aleatorizado	322 (162)	Militares mayores de 18 años, < 16 semanas de gestación	Seis veces más posibilidades de recibir una atención prenatal más adecuada. Mayor satisfacción con su atención (p < 0.001). Mayor número de visitas prenatales en promedio (10.31 visitas vs. 8.56) y, de aquellas en ateción prenatal en grupo, sólo un 12.9% tenían menos de 9 visitas vs. 46.7% de aquellas en atención individual p < 0.005, OR:5.92 IC 3.21-10.91)
Ford, 2002	Ensayo clínico aleatorizado	282 (165)	Adolescentes de 13-21 años	Mujeres en atención prenatal en grupo tenían menos tasas de recién nacidos con bajo peso (6.6% vs. 12.5%, p 0.08)

Los resultados perinatales más estudiados han sido: parto pre-término^6 , 16 , 17 , 21^ , bajo peso al nacer^6 , 16 , 17 , 21 , 22^ , tasa de admisión a Unidades de Cuidados Intensivos^6 , 16 , 21^ , talla para la edad gestacional^21 , 23^ y mortalidad neonatal^21^ . Los resultados reportados no fueron concluyentes para señalar alguna superioridad de la atención prenatal en grupo ( Tabla 2 ). Por otro lado, ningún estudio ha reportado que el modelo tenga resultados perjudiciales para la madre o el recién nacido^16^ .

El modelo grupal logró mayor efectividad para resultados muy específicos (conocimiento, planificación familiar, y uso de servicios). Al ser un modelo claramente centrado en la mujer, quizás sean estos los resultados con mayores probabilidades de ser alcanzados. Otros requieren de cambios más estructurales en el sistema de salud y en otros determinantes sociales, más allá de solo un cambio en el modelo de atención. Futuras evaluaciones deberán fortalecer la evidencia sobre los efectos en satisfacción, autoeficacia, empoderamiento y mejoras en la experiencia durante el embarazo. Estos constituyen resultados más inmediatos a ser alcanzados, de acuerdo con los principios de los modelos de atención centrados en las usuarias^27^ .

## EVIDENCIA DE LA IMPLEMENTACIÓN DE MODELOS DE ATENCIÓN PRENATAL EN GRUPO EN DISTINTOS CONTEXTOS: RETOS Y POTENCIALES SOLUCIONES

La implementación del modelo de atención prenatal en grupo cuenta con retos que pueden ser analizados desde la perspectiva de la oferta de los servicios de salud y de la población usuaria o demandante de dichos servicios.

Los retos de la oferta son aquellos relacionados con el personal que implementará el modelo^6^ , los recursos materiales e insumos físicos^5 , 6 , 17^ y aquellos organizacionales, propios de las instituciones o normativas nacionales de provisión de servicios de salud^13^ ( Tabla 4 ). Estos retos tienen un potencial impacto en los niveles de aceptabilidad que los prestadores de salud refieren al adoptar el modelo^6^ . Los mayores retos de la oferta se centran en la parte estructural de los servicios de salud. Si bien los requerimientos de espacios físicos son decisivos para la implementación del modelo, las evidencias señalan alternativas factibles de solución^12^ .

**Tabla 4:** Retos, recomendaciones y potenciales soluciones para implementar el modelo de atención prenatal en grupo en distintos contextos, 2002–2018.

Retos			Recomendaciones, potenciales soluciones y experiencia en México	Elemento esencial involucrado (Tabla 1)
Oferta	Personal a implementar el modelo	Perfil	Equipo multidisciplinario: puede incluir parteras profesionales, personal médico, personal de enfermería, educador en salud. Es importante que se sientan cómodos con un cambio de paradigma y tengan una actitud positiva para implementar un nuevo modelo	No explícitamente identificado
			Personal médico, de enfermería y de promoción de la salud	
		Número	Uno, dos o tres facilitadores por grupo	7
			Dos a tres facilitadores por grupo	
		Continuidad de la atención	Mismo equipo de facilitadores en cada sesión	6
			Capacitar a dos equipos de facilitadores en el mismo centro de salud que tengan la capacidad de dar seguimiento a las mujeres en caso necesario	
		Capacitaciones (costo y logística)	Financiamiento y apoyo del área directiva para la capacitación de los sitios	No explícitamente identificado
			Programar capacitaciones con varios centros de salud en conjunto. Formar un equipo de “capacitadoras” locales que puedan replicar los talleres en el país	
		Lograr un estilo facilitador y una relación más horizontal entre el personal de salud y la usuaria	Durante la capacitación reforzar que la información debe ser facilitada entre todo el grupo, más que proporcionada únicamente por el personal de salud. Los facilitadores guían pero no controlan la plática. Los facilitadores deben evitar vestimentas que denoten una jerarquía (por ejemplo batas). Las mujeres se involucran en medir algunos parámetros de salud	2, 3, 5, 8, 9
			Tener durante todas las sesiones, al menos del primer grupo, a una persona que esté apoyando en reforzar la nueva dinámica y, al final de la sesión, realizar con los facilitadores un recuento de lo que sucedió en la sesión y lo que puede mejorar	
	Recursos materiales e insumos físicos	Espacio físico	Buscar un espacio semi-privado en la clínica que permita al mismo tiempo la presencia de 10-12 personas en un círculo y que en el mismo espacio pero atrás del círculo se pueda instalar un espacio para llevar a cabo revisiones físicas	1, 5, 8
			Utilizar cuartos de descanso, salas de espera en horarios menos concurridos, un lugar abierto bajo los árboles, estacionamiento, la casa de una persona del grupo, una tienda de campaña y de preferencia temprano en la mañana o ya por la tarde para evitar temperaturas altas. Buscar lugares comunitarios como iglesias u otros	
			Buscar un lugar cercano a la clínica (espacio del gobierno, iglesia, centro ejidal) semi-privado, gratuito, que permita un traslado corto del personal de salud y del material a utilizar, y que sea accesible para las mujeres	
		Adaptación del material y costos recurrentes del mismo	Adaptar el material tomando en cuenta algunos aspectos como el estatus socioeconómico, la raza, la religión, el lenguaje y la cultura	4
			Apoyarse de literatura del lugar, expertos y locales para adaptar el material	
			Trabajar en la adaptación del material con expertas y un equipo multidisciplinario que conozca a la población y pensar en dinámicas que no requieran de compras recurrentes. Identificar el material recurrente (ejemplo: hojas de papel, pilas para esfigmomanómetros) y realizar un plan para solicitar apoyo a nivel estatal o federal	
	Organizacionales	Gerencia y coordinación al interior de las unidades / clínicas	Involucrar a los encargados de las clínicas de manera directa en el proyecto haciendo énfasis en la importancia del apoyo y comprensión de todas las personas de la clínica para solución de situaciones inesperadas y flexibilidad durante los días necesarios para llevar a cabo la atención prenatal en grupo	No explícitamente identificado
		Normativas nacionales	Adaptar los materiales siguiendo las normativas nacionales, diseñando un currículum con los temas que refiere la Norma Oficial Mexicana que las mujeres embarazadas deben ver durante sus consultas prenatales; pero siendo lo suficientemente flexible para incluir temas emergentes de interés para las mujeres	4
Demanda	Usuarias del modelo	Reclutamiento	Reclutar participantes con semanas similares de gestación	7
			Que todo el personal de la clínica conozca los beneficios del modelo para que puedan invitar a las mujeres a participar. Que las personas que tienen un contacto inicial con las mujeres embarazadas estén capacitados en cómo reclutar a las mujeres. Que la persona que reclute a las mujeres sea un personal de salud al que las mujeres le tengan confianza Tener folletos o material que ayuden durante el reclutamiento para explicar de lo que se trata el modelo Que la pareja esté presente durante el reclutamiento	
			Identificar sitios con suficiente volumen de mujeres embarazadas	
		Retención	Retención de participantes con semanas similares de gestación	6
			Recordatorios por medio de medios electrónicos sobre la próxima sesión	
		Lenguaje y nivel educativo de las usuarias	Adaptar los materiales al nivel educativo y lenguaje de la población clave	4
			Contar con facilitadores capacitados que hablen el idioma de la población Llevar a cabo dinámicas en las que no requiera que las mujeres lean sino solamente hablen; usar dinámicas que incluyan canciones del lugar Llevar a cabo la autoevaluación con apoyo de los facilitadores siempre	
			Identificar material y dinámicas sencillas y adaptar el material con ayuda de expertas y un equipo multidisciplinario que conozca a la población; adaptar juegos populares del lugar	
		Privacidad	Utilizar música y tener un espacio para la revisión clínica atrás del círculo un poco alejado	1
			Utilizar un biombo o cortina para separar el espacio de la revisión clínica	
			Utilizar un biombo para separar el espacio de la revisión clínica	
		Que las mujeres se apeguen a las reglas establecidas en el grupo (horario, llevar o no acompañantes)	Dejar claro en la primera sesión las reglas del grupo y permitir que las mujeres cambien aquellas que no se adaptan a sus necesidades	6,7
			En algunos casos permitir acompañantes durante todas las sesiones, incluyendo niños	
			Flexibilidad para iniciar la sesión 15-20 minutos después, o para permitir que algunas mujeres vayan llegando a la sesión e incorporarlas a las actividades. Permitir que lleven a sus acompañantes aunque no sea de manera consistente, sobre todo sus parejas, ya que a veces pueden tener dificultad en el trabajo para solicitar permiso y acudir a todas las sesiones	

NOTA: En el apartado de recomendaciones, potenciales soluciones y experiencia en México, en blanco, se describe la recomendación para implementar el modelo; en gris, la solución descrita en la literatura; y en azul, nuestra experiencia en México.

La atención prenatal en grupo se ha implementado en distintas poblaciones en EEUU, por ejemplo: mujeres afroamericanas^5 , 6 , 17 , 22^ , militares^5 , 6 , 16 , 17^ , y algunas poblaciones que podrían ser de interés para países en Latinoamérica como: adolescentes^5 , 6 , 26^ , latinas o hispanas^5 , 6 , 16^ , mujeres con diabetes^5 , 6 , 26^ , y mujeres de bajos ingresos^5 , 6 , 11 , 16 , 26^ . Sin embargo, los retos desde la perspectiva de la demanda son escasos. Algunos incluyen: que las mujeres quieran participar en un nuevo modelo de atención, que sigan acudiendo a sus consultas^6^ , su lenguaje y nivel educativo; y que ajusten sus horarios y actividades, dado que las sesiones grupales tienen mayor duración que la consulta individual^12^ . Otro de los retos reportados es que las mujeres tengan preocupaciones relacionadas con la privacidad^5^ . El enfoque del modelo centrado en la usuaria también podría explicar los escasos retos identificados desde la perspectiva de la demanda. Este tipo de atención se ha descrito también vinculada con una percepción de derechos, influyendo positivamente en su aceptabilidad, por parte de la demanda^27^ .

Algunos de los retos pueden ser dependientes del contexto o cultura, para los cuales se han planteado algunas soluciones muy específicas ( Tabla 4 ). A pesar de esto, su difusión y discusión entre actores interesados, tanto del ámbito clínico, como académico y con tomadores de decisiones, puede ser de utilidad para futuros esfuerzos de implementación del modelo en Latinoamérica.

## LA EXPERIENCIA MEXICANA DURANTE LA ADAPTACIÓN E IMPLEMENTACIÓN DE UN MODELO DE ATENCIÓN PRENATAL EN GRUPO

Investigadoras del Instituto Nacional de Salud Pública de México (INSP) hemos adaptado e implementado el modelo *CenteringPregnancy* en el sector público del sistema de salud mexicano durante 2016–2018. Como en muchos países, este sector cuenta con retos particulares, sobre todo en infraestructura, financiamiento y organización.

Resumimos nuestra experiencia de la adaptación e implementación del modelo en la Tabla 5 . Una descripción más detallada del proceso de adaptación se puede consultar en un artículo previo^13^ . Dos retos y sus respectivas soluciones o recomendaciones se destacan (Tablas 4 y 5)^28^ .

**Tabla 5:** Experiencia de adaptación e implementación del modelo de Atención Prenatal en Grupo. México, 2016–2018.

Adaptación e implementación del modelo en México	Descripción		
Fases del estudio de factibilidad	Constó de 6 fases y lo llevamos a cabo de junio de 2016 a agosto de 2018. 1. **Involucramiento de la Secretaría de Salud y capacitación del equipo mexicano** . Negociamos la implementación del modelo con tomadores de decisiones de dos estados de México (Morelos e Hidalgo). Al mismo tiempo, dos integrantes del equipo de investigación se capacitaron como facilitadoras en un taller intensivo del modelo *CenteringPregnancy* en Estados Unidos. Durante esta y las siguientes fases, contamos con el apoyo de una experta internacional en el modelo. 2. **Adaptación del modelo** CenteringPregnancy **al contexto mexicano** . Integramos un currículo con base en los lineamientos de la consulta prenatal de la Secretaria de Salud, la percepción de las gestantes y expertos en el tema de salud materna. También adaptamos recursos materiales e insumos físicos. 3. **Selección de sitios participantes** . Realizamos estudios de pre-factibilidad en varios centros de atención de primer nivel en los estados de Morelos e Hidalgo, identificando elementos estructurales (cantidad de consultorios, personal de salud, espacios disponibles, etc.) así como el volumen de la demanda para la atención prenatal (número de consultas prenatales en el último año). Considerando esta información y apoyo de la experta internacional, seleccionamos los centros de salud donde se implementó el modelo. 4. **Capacitación del personal de salud de los centros de salud.** Capacitamos a los proveedores de salud (personal de enfermería, personal médico y de trabajo social) para asumir el rol de facilitadores del modelo. Esta fase también ayudó a que dos integrantes del equipo se consolidaran como capacitadoras del modelo. 5. **Estudio Piloto.** En esta fase comenzamos la implementación del modelo en dos sitios, uno en cada estado. Entregamos el material necesario para la implementación y dimos asesoramiento para el reclutamiento inicial de mujeres. El equipo de investigación acompañó de manera directa para la solución de problemas. Adecuamos los procesos, los instrumentos, la temática, la metodología y los aspectos logísticos en conjunto con la experta internacional. 6. **Implementación** : Tomando en cuenta todas las adaptaciones de las fases previas, ampliamos la implementación del modelo a la totalidad de los sitios seleccionados.		
Estados donde implementamos el modelo	Los estados seleccionados fueron: Morelos e Hidalgo. Las Jurisdicciones Sanitarias^*^: Morelos- Jurisdicción 1. Hidalgo- Jurisdicción 3.* Los sitios de implementación pertenecen a la Secretaría de Salud (Servicios Estatales de Salud) que brinda servicios a la población que no cuenta con ningún tipo de seguridad social, es decir, la población más vulnerable del país que no cuenta con un trabajo en el sector formal de la economía.		
Número y sitios donde implementamos el modelo	Sitios de implementación en Morelos (3): Centros de Salud Acatlipa, Temixco y Emiliano Zapata Sitios de implementación en Hidalgo (1): Centro de Salud de Tula		
Número y perfil de facilitadores capacitados por sitio	Un total de 23 proveedores de salud fueron capacitados a lo largo de todo el estudio (3 capacitaciones de 2 días cada una); de estos, 15 participaron como facilitadores de grupos en diferentes centros:		
	Sitio	Total de facilitadores	Perfil de facilitadores
	Acatlipa, Morelos	3	1 médica, 1 médico y 1 enfermera
	Temixco, Morelos	2	1 médica y 1 enfermera
	Emiliano Zapata, Morelos	5	1 médica, 1 médico, 2 enfermeras y 1 promotora de salud (Lic. Psicología)
	Tula, Hidalgo	5	1 médica, 1 médico, 1 enfermera, 1 enfermero y 1 trabajadora social
Número de grupos implementados	Se llevaron a cabo un total de 11 grupos en ambos estados (siete en Morelos y cuatro en Hidalgo): • Morelos: Acatlipa (3 grupos), Temixco (2 grupos), Emiliano Zapata (2 grupos) • Hidalgo: Tula (4 grupos)		
Número de gestantes captadas	El total de gestantes captadas para los 11 grupos fue de 142 mujeres.		
Número de gestantes que concluyeron el modelo	El 76.0% (108) terminó su atención prenatal en grupo.		
Edad gestacional al inicio del modelo	Promedio de edad gestacional al inicio de su atención prenatal en grupo: 23.0 semanas de gestación. Rango: 21.9 – 24.1.		
Promedio de visitas prenatales	5.5 visitas por mujer		
Porcentaje de mujeres con al menos 5 consultas prenatales	El total de mujeres que cumplieron con lo establecido en la Norma Oficial Mexicana sobre atención prenatal fue de 115 (81.0%)		

* Se refiere a una unidad técnico-administrativa desconcentrada de los Servicios Estatales de Salud, con capacidad para la planeación, administración, dirección, operación y evaluación de los recursos para “otorgar atención médica a población no asegurada, con el propósito de conducir adecuadamente las acciones del sector en su área de influencia”^28^.

Es necesario hacer una selección de unidades guiada por criterios puntuales que faciliten la implementación. Entre estos criterios destacamos: 1) contar con el espacio físico adecuado para la atención grupal, permitiendo la reunión de alrededor de 15 personas; 2) contar con suficiente personal de salud en cada sitio que permita capacitar a más de un equipo de facilitadores, tanto para el seguimiento de los grupos, como para el reclutamiento de las participantes; y 3) contar con el apoyo del área directiva y administrativa de la unidad, lo cual es clave debido al tiempo destinado a actividades de logística y organización antes, durante y posterior a las sesiones.

El segundo gran reto es el cambio de paradigma sobre cómo transmitir la información y brindar atención durante el embarazo, debido a la cultura médica jerárquica prevaleciente. La presencia de una persona del equipo de investigación en las sesiones permitió apoyar y reforzar la adopción de este nuevo estilo facilitador y dar retroalimentación una vez terminada la sesión. Una posible limitante de esta solución es que no siempre podría monitorearse la implementación. Sin embargo, se podrían establecer estrategias como capacitaciones intermedias de reforzamiento durante el tiempo entre la etapa de “adaptación” y la de “adopción”. La primera etapa permite ajustar el modelo al contexto local, y la segunda (más difícil) se refiere a que el personal de salud y las usuarias lo adopten como una práctica aprendida e internalizada.

La evidencia preliminar mostró factibilidad para la implementación del modelo en México, siempre que se siga un proceso de pasos escalonados que propicien el convencimiento de las autoridades, capacitación y sensibilización del personal de salud y adecuada selección de las unidades participantes.

## CONSIDERACIONES FINALES

La calidad y continuidad de la atención prenatal sigue siendo un reto en países hispanohablantes y latinoamericanos^29^ , sobre todo en poblaciones en condiciones de vulnerabilidad^30^. La atención prenatal en grupo representa una oportunidad en estos grupos poblacionales: es un modelo centrado en la usuaria, que mejora indicadores de salud materna, logrando mayor apego al control prenatal, incrementando los conocimientos de las gestantes y el uso de servicios de planificación familiar postparto.

El enfoque que caracteriza al modelo, en el que la mujer es el centro de la atención, logra motivar la participación e interés de la usuaria. Además, favorece una experiencia positiva durante el embarazo, con una atención respetuosa, basada en una visión de derechos, alto nivel de participación e involucramiento de la gestante en el cuidado de su salud. Consideramos eso algo sumamente novedoso y valioso como alternativa a los modelos ya existentes. A pesar de que no hay resultados concluyentes en relación a la satisfacción de las usuarias, esperamos que próximos estudios profundicen más en este punto dado la naturaleza del modelo.

Se requiere desarrollar estudios con diseños rigurosos y a mayor escala, tanto en el contexto nacional como en países similares al nuestro. Esos estudios pueden contribuir para generar evidencia que sustente las recomendaciones referentes a la implementación de este tipo de modelos y que permitan, eventualmente, evaluar no solo factibilidad sino sostenibilidad.
